# Female heterogamety in Madagascar chameleons (Squamata: Chamaeleonidae: *Furcifer*): differentiation of sex and neo-sex chromosomes

**DOI:** 10.1038/srep13196

**Published:** 2015-08-19

**Authors:** Michail Rovatsos, Martina Johnson Pokorná, Marie Altmanová, Lukáš Kratochvíl

**Affiliations:** 1 Department of Ecology, Faculty of Science, Charles University in Prague, Prague, 128 44, Czech Republic; 2 Institute of Animal Physiology and Genetics, The Academy of Sciences of the Czech Republic, Liběchov, 277 21, Czech Republic

## Abstract

Amniotes possess variability in sex determining mechanisms, however, this diversity is still only partially known throughout the clade and sex determining systems still remain unknown even in such a popular and distinctive lineage as chameleons (Squamata: Acrodonta: Chamaeleonidae). Here, we present evidence for female heterogamety in this group. The Malagasy giant chameleon (*Furcifer oustaleti*) (chromosome number 2n = 22) possesses heteromorphic Z and W sex chromosomes with heterochromatic W. The panther chameleon (*Furcifer pardalis*) (2n = 22 in males, 21 in females), the second most popular chameleon species in the world pet trade, exhibits a rather rare Z_1_Z_1_Z_2_Z_2_/Z_1_Z_2_W system of multiple sex chromosomes, which most likely evolved from W-autosome fusion. Notably, its neo-W chromosome is partially heterochromatic and its female-specific genetic content has expanded into the previously autosomal region. Showing clear evidence for genotypic sex determination in the panther chameleon, we resolve the long-standing question of whether or not environmental sex determination exists in this species. Together with recent findings in other reptile lineages, our work demonstrates that female heterogamety is widespread among amniotes, adding another important piece to the mosaic of knowledge on sex determination in amniotes needed to understand the evolution of this important trait.

Chameleons (family Chamaeleonidae) are well-known, highly distinctive lizards characterised by their unique morphological and physiological traits, such as independently movable stereoscopic eyes, projectable, ballistic tongue, prehensile feet, and the notable ability in many species to change the colour of their skin. Currently, 200 extant species are recognized, distributed in Africa, Madagascar, Southern Europe and Southern Asia^1^. For a lizard family, chameleons have a relatively recent origin, with the basal split dated at approximately 65 million years ago^2^.

Despite the popularity of chameleons among herpetologists, private breeders and hobbyists, their sex determination has remained largely unstudied. In general, reptiles show a wide diversity of sex determination systems, including female heterogamety, male hetorogamety and environmental, particularly temperature-dependent, sex determination^3^,^4^. Chameleons are an interesting group for the study of the evolution of sex determination as their sister lineage, dragon lizards (Agamidae), possesses either female heterogamety or environmental sex determination^3^,^5^,^6^, while the next outgroup, iguanas (Pleurodonta), are typified by male heterogamety and a high evolutionary stability of sex chromosomes^7^,^8^,^9^. Although temperature-dependent sex determination has been anecdotally reported for several chameleon species, e.g., *Furcifer pardalis*, *Chamaeleo chameleon*, *C. calyptratus*, the evidence for it is not supported by controlled experiments^6^,^10^,^11^. Moreover, where such experiments were performed, they showed that hatchling sex ratios do not deviate significantly from 1:1 at different constant incubation temperatures in *F. pardalis*, nor in *C. calyptratus*^10^,^11^. The observation of equal sex ratios across constant incubation temperatures led to the conclusion that chameleons possess genotypic sex determination^6^,^11^.

Surprisingly, chameleons are relatively poorly studied cytogenetically and karyotypes are known for only 50 out of about 200 extant species. They possess karyotypes with chromosomal numbers varying from 2n = 20 to 2n = 36 (ref. 12). However, all known karyotypes have only been studied using exclusively “classical” cytogenetic techniques, and molecular cytogenetic approaches have not yet been applied. In addition, sex chromosomes have not been described in any species, largely as few of these studies examined both males and females, which precluded the discovery of sex chromosomes. The only known exception has come from Gordon (pers. comm. reported in ref. 12) who suggested the existence of a female heterogametic system in *Bradypodion ventrale*, in response to the observation of the different chromosomal number existing between males (2n = 34) and females (2n = 35). Unfortunately though, to our knowledge, neither karyotypes of this species, nor any other additional information which would enable the testing of sex-linkage of the polymorphism in chromosomal numbers, has been published.

This lack of comprehensive data prompted us to apply both classical (Giemsa staining, C-banding) and molecular (comparative genomic hybridization [CGH], fluorescence *in situ* hybridization [FISH]) cytogenetic approaches to reveal the karyotype and particularly the sex chromosomes of two species of chameleons from the genus *Furcifer*. The panther chameleon (*Furcifer pardalis*) and Malagasy giant chameleon (*Furcifer oustaleti*) are endemic to Madagascar and adjacent islands, with the panther chameleon also being recently introduced to Reunion Island and Mauritius, and the Malagasy giant chameleon becoming established near Nairobi in Kenya^13^,^14^,^15^,^16^. In Madagascar, both species are common in lowlands, occupying a variety of habitats, such as scrub and forest, and have so far survived the increased levels of habitat exploitation and degradation caused by human activities^17^,^18^. The chameleons of the genus *Furcifer* possess karyotypes with chromosomal numbers varying between 2n = 22 and 2n = 28 (ref. 12) and karyotypes with 2n = 22 chromosomes were reported for both *F. pardalis* and *F. oustaleti*^19^,^20^,^21^. Panther chameleons exhibit high variation in colouration with several geographically distinct colour morphs^22^. In addition, due to their variegated colouration, relatively easy care and reproduction in captivity they are very popular pets in North America and Europe with large numbers being bred commercially^16^,^22^.

## Results

The 664 bp sequence corresponding to the 5′ end of the mitochondrial locus cytochrome c oxidase I (COI), was successfully amplified and sequenced in the studied chameleons. The haplotype analysis of our dataset in DnaSP v5.10.1 revealed five haplotypes for *F. pardalis* and two for *F. oustaleti*, none of which previously reported in the GenBank (Table 1). The pooled sequences of COI from both our work and those derived from the GenBank^23^ show that *F. pardalis* altogether demonstrates a low mean genetic variation of 1.5%, while *F. oustaleti* show a relative high genetic variation of 5.9%.

**Figure 1:**
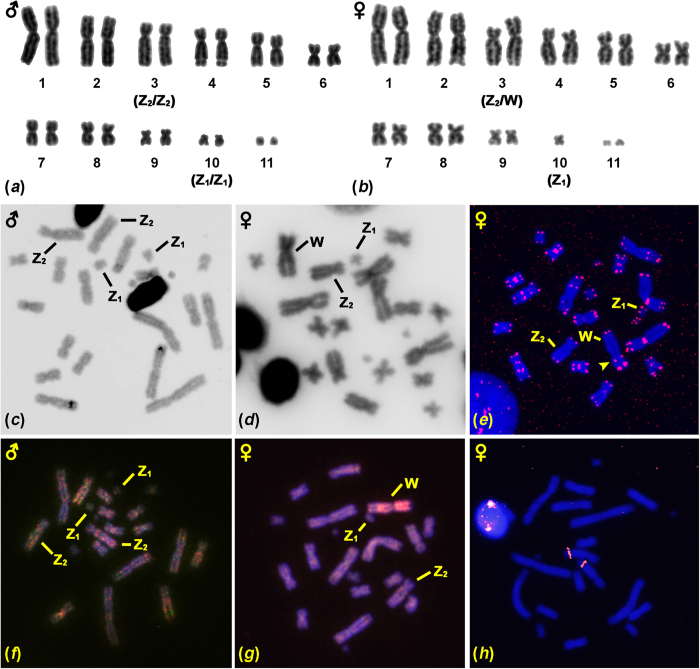
Cytogenetic analysis of *Furcifer pardalis*. Giemsa stained karyotype (**a**,**b**), C-banding (**c**,**d**), topology of telomeric motifs (**e**), comparative genomic hybridization (male DNA labelled with green, female DNA with red) (**f**,**g**) and topology of rRNA genes (**h**). The sex, sex chromosomes and the telomere signal on the potential fusion point in the neo-W chromosome are indicated.

In *F. pardalis*, all 7 males share the karyotype of 2n = 22, however all 6 females possess 2n = 21. The linkage of different chromosomal numbers to sex is statistically highly significant (p = (1/2)^13^ = 0.00012) [we studied 13 individuals and probability that it would be found in a given sex just by chance is ½], which supports the identification of the chromosomes differing between males and females as the sex chromosomes. In females, the q-arm of one chromosome of the third largest chromosomal pair (FPA3) is always longer than its counterpart and this larger chromosome is not present in the male karyotypes. This chromosome seems to be partially heterochromatic, based on C-banding and shows strong female-specific genetic content according to CGH (Fig. 1d,g). Moreover, there is a single small unpaired metacentric chromosome (FPA10) in the female karyotypes (Fig. 1b). On the contrary, in males, the third largest pair of chromosomes (FPA3) is homomorphic and the FPA10 pair consists of two homomorphic, metacentric chromosomes (Fig. 1a). This situation corresponds to the Z_1_Z_1_Z_2_Z_2_/Z_1_Z_2_W sex determination system. As we did not notice any differences in karyotypes among individuals with different haplotypes and from separate regions/colour morphs, the differences between sexes in chromosome number and morphology cannot therefore be attributed to geographic variation in karyotype or to the hybridization of different karyotype races.

**Figure 2:**
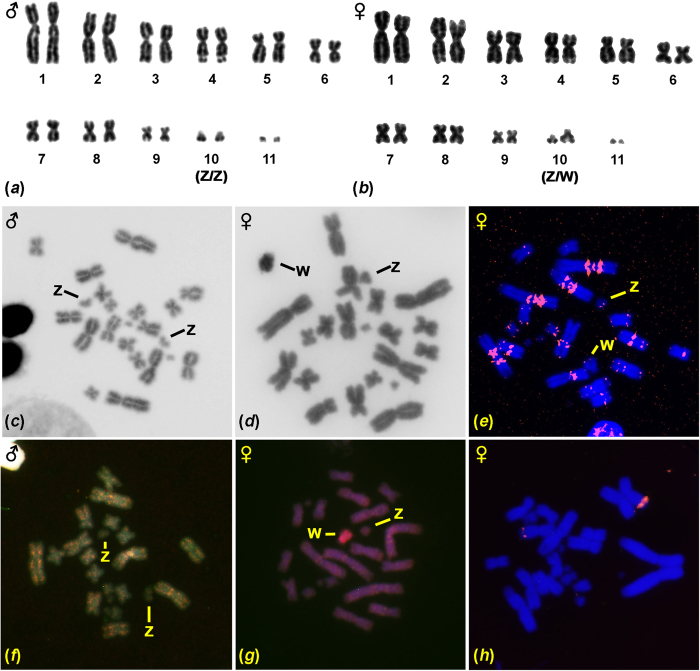
Cytogenetic analysis of *Furcifer oustaleti.* Giemsa stained karyotype (**a**,**b**), C-banding (**c**,**d**), topology of telomeric motifs (**e**), comparative genomic hybridization (male DNA labelled with green, female DNA with red) (**f**,**g**) and topology of rRNA genes (**h**). The sex of the studied individual and sex chromosomes are indicated.

The karyotype of both sexes of *F. oustaleti* consists of 2n = 22. However, we noticed heteromorphy of the second smallest chromosomal pair (FOU10) in the female karyotype (Fig. 2b), while both chromosomes of FOU10 are of equal size and shape in males (Fig. 2a). The larger, female-specific chromosome of FOU10 is strongly heterochromatic as visualized by C-banding and contains female-specific sequences as revealed by CGH (Fig. 2d,g). Although our sample size in *F. oustaleti* is small, the similarity of karyotypes in both studied species, the heterochromatic content of the submetacentric chromosome, and its distinctness in CGH enable us to conclude that the submetacentric chromosome FOU10 in females can be assigned as the W sex chromosome.

In addition to the expected terminal topology, FISH with the probe specific for the telomeric motif revealed extended interstitial telomeric repeats (ITRs), in both species (Figs 1e and 2e). In *F. pardalis*, telomeric-like sequences were detected in the centromeric regions of six chromosomal pairs (1, 4–8) and interstitial positions in three chromosomal pairs (1, 2, 4) and in the W chromosome (Fig. 1e). In *F. oustaleti*, telomeric-like sequences were detected in the centromeric regions of four chromosomal pairs (1–4) and in the pericentromeric and interstitial positions of eight chromosomal pairs (1–8) (Fig. 2e).

In both species, we identified a strong signal of the rRNA probe in the fourth largest chromosomal pair localized in the region of the secondary constriction (Figs 1h and 2h).

## Discussion

Our results provide the first unequivocal evidence of the presence of sex chromosomes in any species of chameleons. We can conclude that chameleons of the genus *Furcifer* possess female heterogamety with well-differentiated sex chromosomes. In earlier studies^20^,^21^, the differences in chromosome number between males and females in *F. pardalis* were not noticed. We think that the discrepancy can be attributed to the limitations of microscopy and chromosome preparation techniques at the time of the earlier studies (see also ref. 24 for another recent correction of the original cytogenetic data). In addition, neither the sex, nor the number of studied chameleon individuals were indicated and perhaps mainly male individuals were karyotyped and in several cases, testicular tissue was used for metaphase preparations^19^,^20^,^21^, and thus, the authors of the earlier studies were not able to identify the neo-W chromosome. The previous unpublished record by Gordon (pers. comm. reported in ref. 12) suggested the existence of female heterogamety in *Bradypodion ventrale* as well. As there is no reliable evidence for environmental sex determination in chameleons^6^,^11^ and taking into account that sex chromosomes are usually rather conserved in many main lineages of amniotes^3^,^4^,^25^, female heterogamety might be widespread and conserved in chameleons, at least in the *Furcifer-Bradypodion* clade. However, additional molecular cytogenetic studies with extended sampling of chameleon genera are needed to prove this speculation.

*Furcifer pardalis* possesses the multiple sex chromosome system Z_1_Z_1_Z_2_Z_2_/Z_1_Z_2_W. These multiple sex chromosomes likely evolved through the fusion of the ancestral W with an autosome to form the neo-W sex chromosome. The original Z remained unchanged and the other half from the original autosomal pair can be assigned as the neo-Z sex chromosome. The W chromosome of *F. oustaleti* is highly heterochromatic, which is the typical situation in many squamate lineages with highly differentiated Z and W chromosomes^5^,^24^,^26^,^27^,^28^. The W chromosome of *F. oustaleti* is almost two-fold bigger in size than its Z counterpart, a situation not often reported in squamates^29^,^30^, but similar cases were reported in varanids, agamids and geckos^26^,^31^,^32^,^33^. We assume that the W chromosome of *F. oustaleti* increased in size due to the amplification of repetitive elements, a process often occurring in the X and Y heterochromatic blocks of rodents and reported also in the W chromosome in a lizard^26^,^34^,^35^.

The neo-W in *F. pardalis* is only partially heterochromatic and ITRs were detected in the putative fusion point which may be remnants of the ancestral chromosome telomeres (Fig. 1e). The fusion of an unpaired heterochromatic sex chromosome to an autosome usually leads to the rapid spread of heterochromatin to the previously autosomal parts of newly formed neo-sex chromosomes^36^,^37^, which we speculate is probably also the case in *F. pardalis*. The colonization of the previously autosomal region of the neo-W sex chromosomes by repetitive elements (e.g., transposons) already accumulated on the W-specific part of the ancestral W would explain the extensive spread of female-specific content across the neo-W chromosome, as revealed by CGH (Fig. 1g). Further comparative work is required to verify these assumptions in order to fully reconstruct the evolution of the sex and neo-sex chromosomes of chameleons.

Among amniote vertebrates, the emergence of multiple neo-sex chromosomes is quite frequent in lineages with male heterogamety such as mammals or iguanas. In comparison however, it is exceptional in lineages with female heterogamety, being reported only in lacertid lizards and elapid snakes^38^. Furthermore, it appears that multiple neo-sex chromosomes have never evolved in the well-studied and highly diversified clade of birds which also exhibits female heterogamety. Previously, we hypothesized that this difference might be explained by the variable effect of the formation of multiple neo-sex chromosomes on sex ratios under female meiotic drive. We suggested that Y chromosomes are never involved in female meiosis and so their rearrangements are therefore sheltered against female meiotic drive. On the other hand, Z and W sex chromosomes are always involved in female meiosis. Therefore, rearranged Z or W sex chromosomes may have selectively preferred access to the egg nucleus or to the nucleus of a polar body by female meiotic drive, which would lead to a biased sex ratio. Following our model, rearrangements of Z and W should be penalized by selection for a balanced sex ratio. Being easily bred in captivity, *F. pardalis* with their multiple neo-sex chromosomes and close relatives with ordinary ZZ/ZW chromosomes, might be a wonderful system with which to explore the evolution of multiple neo-sex chromosomes under female heterogamety, which may break the suggested constraint imposed by female meiotic drive.

*In situ* hybridization experiments show that the rRNA genes are located in the secondary constriction region of the fourth chromosomal pair in both species. The localization of such genes close to or inside these regions is commonly reported in several lizard lineages, including agamids^39^,^40^, lacertids^41^ and varanids^42^. Also, the occurrence of telomeric-like sequences distant from terminal positions has been recorded in several vertebrate species^43^, and may be strongly accumulated even in the heterochromatic content of sex chromosomes^26^,^33^. Interstitial telomeric repeats are often considered remnants of chromosomal rearrangements^44^. The family Chamaeleonidae demonstrates significant chromosomal number variation among species, ranging from 2n = 20 to 2n = 36 (ref. 12). Taking into account that both *F. pardalis* and *F. oustaleti* possess karyotypes with a low chromosomal number (2n = 22), we speculate that the observed ITRs probably correspond to the chromosomal fusions which occurred during the formation of their derived karyotypes.

The aim of DNA barcoding is the rapid and accurate molecular identification of species or geographic origin of individuals by comparing the studied samples with the sequences deposited in reference databases^45^,^46^,^47^. The recent boost of DNA barcoding in Madagascar herpetofauna^23^ and the formation of the “Cold Code” consortium^48^ as part of the global initiative to barcode amphibians and non-avian reptiles has promoted the COI gene as a recommended marker for reptile identification, allowing researchers from various scientific fields to cross-reference the studied material regardless of potential taxonomic changes (for a recent example see ref. 49). In still poorly studied biodiversity hotspots, such as Madagascar, many species demonstrate populations with high genetic distances, which are currently regarded as intraspecific polymorphism but probably correspond to species complexes, which could be distinguished as independent species after proper taxonomic analysis (see ref. 23, for identification of more than 40 possible cryptic reptile Madagascan species using DNA barcoding of COI sequences). Both *F. pardalis* and *F. oustaleti* probably form a species complex too^16^,^18^,^50^. The very recent study demonstrates clear phylogeographic structure among populations/colour morphs of *F. pardalis*^16^, however, the genetic distances among them are rather low. Our individuals originated from the pet trade and their area of origin presented in the Table 1 should be treated with caution. The geographic origin of our specimens can be confirmed in future based on known phylogeographic pattern of COI sequences. Nevertheless, for our cytogenetic analysis, it is important that we found the fusion leading to neo-W sex chromosome across females of *F. pardalis* from different colour morphs and with different mtDNA haplotypes. Therefore, we can conclude that the emergence of the fused situation is of older origin, it is probably widespread in this species and it cannot be attributed to exceptional karyological nature of our samples, i.e. we can exclude its emergence by a very recent mutation.

Amniotes possess variability in sex determining mechanisms but as this diversity is only partially known there is still not a full understanding of the evolution of sex determining mechanisms in this crucial group. Sex chromosomes in many important lineages have been discovered only very recently^7^,^8^,^9^,^24^,^25^,^26^,^28^,^33^ and several lineages, including chameleons, have remained surprisingly unstudied in this respect. The discovery of sex chromosomes in chameleons as well as the demonstration of a rare case of multiple neo-sex chromosomes under female heterogamety fills another important gap in our still patchy understanding of the sex determination mechanisms in amniotes.

## Material and Methods

### Sampling

Blood samples were collected from 13 individuals of *F. pardalis* (7 males, 6 females) and a pair of *F. oustaleti* (Table 1), kept at the Zoopark Zájezd, Czech Republic. All specimens were wild-caught, legally imported for the pet trade or their direct progeny. The blood samples were subsequently used for DNA isolation and whole blood cell cultures. In order to test generality of our cytogenetic findings in *F. pardalis*, we cytogenetically examined specimens from 7 colour morphs (Table 1).

### DNA barcoding

A DNA barcoding approach was applied in order to properly identify our specimens and subsequently to provide a genetic identity of studied chameleons, which will allow researchers from various scientific fields to follow the traces of the cytogenetically studied material regardless of potential taxonomic changes^24^,^28^,^51^. Genomic DNA from each individual was extracted using a DNeasy Blood and Tissue Kit (Qiagen, Valencia, CA), and the 5′ fragment of the mitochondrial COI gene^23^,^52^ was amplified by PCR, using either the reptile-specific primers RepCOI-F and RepCOI-R (ref. 23) or the universal primers LCO1490 and HCO2198 (ref. 53). The PCR reaction and cycling conditions are detailed in (ref. 28). The PCR products were sequenced bidirectionally by Macrogen (Korea) and the obtained haplotype sequences deposited in GenBank under the accession numbers KP715537–KP715542 (Table 1). The COI sequences were aligned using CLUSTALW (ref. 54), included in BioEdit v5.0.9 (ref. 55) and subsequently analysed in DnaSP v5.10.1 (ref. 56). Genetic distances among haplotypes were calculated using the Kimura 2-parameter model in MEGA v6.0.5 (ref. 57).

### Chromosomal preparations and staining

Metaphase chromosome spreads were prepared from whole blood cell cultures^26^. Conventional Giemsa staining was applied to chromosomal preparations from all individuals in order to visualize chromosomal morphology. C-banding^58^ was used to reveal the distribution of the constitutive heterochromatin in the genome.

### Comparative genomic hybridization

CGH was applied in order to reveal sex specific differences in metaphases from both sexes, as described in (ref. 26). Briefly, 1 μg of male and 1 μg of female genomic DNA were labelled with biotin-dUTP and digoxigenin-dUTP, respectively, using a Nick Translation Kit (Roche) and then mixed together. Sonicated salmon sperm DNA was added and ethanol-precipitation was carried out overnight. The labelled DNA was resuspended in hybridization buffer (50% formamide, 2× SSC, 10% SDS, 10% dextran sulfate, 1× Denhardt’s buffer, pH 7), denatured at 75 °C for 10 min and chilled on ice for 10 min prior to hybridization. The slides with chromosomal material were subsequently treated with RNase A and pepsin; fixed with 1% formaldehyde; dehydrated through an ethanol series (70, 85 and 100%), denatured in 70% formamide/2× SSC at 75 °C for 3 min, and dehydrated again. Hybridization was performed at 37 °C for two days. Post-hybridization washes were performed three times in 50% formamide/2× SSC at 42 °C for 5 min and in 2× SSC. The slides were incubated in 100 μl of 4× SSC/5% blocking reagent (Roche) at 37 °C for 30 min and then with 100 μl of 4× SSC/5% blocking reagent including avidin-FITC (Vector Laboratories) and anti-digoxigenin rhodamine (Roche) at 37 °C for 30 min. The slides were finally washed in 4× SSC/0.05% Tween 20, dehydrated, air dried, and mounted with Vectashield DAPI (Vector Laboratories).

### Fluorescence *in situ* hybridization with telomeric probe and rRNA gene

The topology on the karyotype of the telomeric motif (TTAGGG)_n_ and the rRNA genes within the genomes were analysed by FISH. The telomeric probe was produced and labelled with biotin in a single PCR reaction using the primers (TTAGGG)_5_ and (CCCTAA)_5_, without a DNA template^59^. The rRNA gene probe was prepared from a plasmid (pDm r.a 51#1) with a 11.5 kb insert, encoding the 18S and 28S ribosomal units of *Drosophila melanogaster*^60^. The probe was labelled with biotin-dUTP, using a Nick Translation Kit (Abbott).

In both cases, the probe was ethanol-precipitated together with salmon sperm DNA, resuspended in hybridization buffer (50% formamide/2× SSC), then denatured at 75 °C for 6 min and chilled on ice for 10 min prior to hybridization. The chromosomal preparations were treated as in CGH. Hybridization was performed at 37 °C overnight, followed by post-hybridization washes with 50% formamide/2× SSC at 42 °C for 5 min (3 times) and 2× SSC for 5 min (3 times). The slides were incubated in 100 μl of 4× SSC/5% blocking reagent (Roche) at 37 °C for 45 min and then with 100 μl of 4× SSC/5% blocking reagent containing avidin-FITC (Vector Laboratories). The fluorescence signal was enhanced and detected using a modified avidin-FITC/biotinylated anti-avidin system (Vector Laboratories). Finally, the slides were mounted with Vectashield DAPI anti-fade medium (Vector Laboratories).

### Microscopy and image analyses

The images were captured on a Provis AX70 (Olympus) fluorescence microscope with a DP30BW digital camera (Olympus) and superimposed with colour using DP Manager imaging software (Olympus). Selected Giemsa stained metaphases were karyotyped using Ikaros karyotyping software (Metasystems).

### Ethical statement

All experiments were performed in accordance with relevant guidelines and regulations. The animal procedures were carried out under the approval and supervision of the Ethics Committee of the Faculty of Science, Charles University in Prague followed by the Committee for Animal Welfare of the Ministry of Agriculture of the Czech Republic. The handling of animals and blood taking was performed by accredited researchers (certificate Nos. CZ 02529, CZ 01223, CZ 02535).

## Additional Information

**How to cite this article**: Rovatsos, M. *et al.* Female heterogamety in Madagascar chameleons (Squamata: Chamaeleonidae: *Furcifer*): differentiation of sex and neo-sex chromosomes. *Sci. Rep.* **5**, 13196; doi: 10.1038/srep13196 (2015).

## Figures and Tables

**Table 1:** Colour morph, haplotype distribution of the COI sequences and number of panther chameleons and Malagasy giant chameleons used in this study.

**Colour morph**	**No. of specimens**		**Haplotypes (GenBank accesion number)**
	**♂**	**♀**	
*Furcifer pardalis*			
1. Ankaramibe		1	—
2. Ambanja	1	1	KP715540
3. Nosy Faly	2	1	KP715541
4. Nosy Mitsio		1	KP715539
5. Ambilobe	2	1	KP715539, KP715542, KP715542
6. Sambava		1	KP715540
7. Reunion Island	2		KP715540
*Furcifer oustaleti*			
8. Tulear	1	1	KP715537, KP715538
